# Constitutive Description of Extra Strengthening in Gradient Nanotwinned Metals

**DOI:** 10.3390/nano11092375

**Published:** 2021-09-13

**Authors:** Wufan Chen, Panpan Wan, Qingkun Zhao, Haofei Zhou

**Affiliations:** 1 Center for X-Mechanics, Department of Engineering Mechanics, Zhejiang University, Hangzhou 310027, China; scoobi@zju.edu.cn (W.C.); panpanwan@zju.edu.cn (P.W.); qkzhao@zju.edu.cn (Q.Z.); 2 State Key Laboratory of Fluid Power and Mechatronic Systems, Zhejiang University, Hangzhou 310027, China

**Keywords:** gradient nanotwinned metals, extra strengthening, constitutive description, twin thickness, grain size

## Abstract

Gradient nanotwinned (GNT) metals exhibit extra strengthening and work hardening behaviors, which endow them impressive potentials in engineering applications. The increased strength is attributed to the dense interactions between dislocations and boundaries in the grain interiors. However, a constitutive model elucidating the extra strengthening effect is currently lacking. Here, we propose a theoretical framework to describe the mechanical response of GNT metals, especially the unusual extra strengthening behavior. The model captures the deformation mechanisms of GNT metals and coincides well with the reported experiment. The constitutive description developed in this work presents a tool to guide the structural design for developing gradient metallic materials.

## 1. Introduction

Inspired by natural gradient structures [1,2,3,4], investigation on gradient nanotwinned (GNT) metals has been an enduring pursuit owing to their appealing potential in engineering applications. The unusual gradient structure can be synthesized using the techniques of electrodeposition or magnetic sputtering [5]. Excellent performances of GNT metals have been reported recently, including extra strengthening, comparable ductility and enhanced impact hardness [6,7,8,9,10,11,12]. Although strength and ductility are always incompatible in conventional metals, GNT metals are able to reconcile this exclusive relation successfully [13,14,15,16,17,18,19]. Recently, remarkable strength even superior to that of the strongest component in GNT metals has been reported [5,20,21]. When applying uniaxial loading parallel to the twin boundaries (TBs), bundles of concentrated dislocations (BCDs) form and interact to sustain the plastic deformation in GNT metals. The BCDs are composed of three types dislocations which are so-called mode I dislocations with Burgers vectors inclined to TBs, mode II dislocations with Burgers vectors parallel to the twin plane, and geometrically necessary dislocations (GNDs) which are developed to allow deformation gradients among different components with different twin plane spacings. Albeit such unique dislocation patterns in the microstructure of deformed GNT metals have been analyzed in detail, quantitative constitutive relation is yet to be proposed to account for the extra strengthening of GNT metals.

Great efforts have been devoted to derive quantitative expressions for the macroscopic response of gradient materials. For instance, Li et al. [22] developed a theoretical model for surface gradient structures which were composed of coarse-grained (CG) metallic metal in core and fine-grained in thin surface, successfully demonstrating the vital role of grain growth in the mechanical performances of the materials. To understand the extra strain hardening in the gradient surface layers, non-uniform deformation lateral surface was considered to achieve an optimized analysis [23]. Zeng et al. [24] using crystal plastic finite element simulations described the cross-sectional stress and strain distribution of gradient nanograined (GNG) metals under uniform deformation, actually eliminating the effects of loading conditions on the unusual gradient strain. It was recognized that the laminated unequal plastic strain was well accommodated by the GNDs in the layer interfaces under loading conditions [10,25]. The induced strain hardening in gradient nanostructures was further modeled based on the density of associated dislocations on the non-uniform lateral surface [26]. Lu et al. [27] obtained a similar dislocation-based mechanism for GNG materials and incorporated the damage evolution and grain growth into the theoretical analysis. Li et al. [28] focused their interest on the crack growth of grain size gradient and established theoretical solution for fatigue behaviors of GNG materials. Based on the deformation mechanism, constitutive modeling for the GNG materials has been investigated intensively coupled with finite element simulations or desired experiments [24,29,30,31,32,33,34]. In addition, bimodal metals with nano/ultrafine grained and CG grained phases were also constitutively modeled accounting for the gradient strain nearby grain boundaries (GBs) [35]. However, the above analyses were performed on GNG materials, rather than on GNT metallic materials. Due to the novel extra strengthening in GNT metals observed in experiment [5], a practical constitutive model is urgent to be developed for clear illustration of the exceptional performance in GNT metals.

To understand the unusual mechanical behaviors of GNT metals, the present work aims to develop a theoretical framework to quantify the extra strengthening inspired by the experimental observations [5]. We first investigated the uniaxial tensile response in homogenous nanotwinned (NT) components according to the experiment on bulk copper samples. The confined layer slip (CLS) model [36] was used to calculate the yield stress and we also accounted for the back stress originated from the impeded dislocation movement nearby GBs. We then discussed the deformation mechanisms that govern the extra strengthening of GNT metals and obtained the extra yield stress by considering the density of BCDs, which was in good agreement with experimental work [5]. The established quantitative model is concise in the plastic flow stage and captures the key factors of the GNT metals, i.e. grain size, twin thickness and the strain gradients in the transition zone.

## 2. Materials and Methods

**Figure 1:**
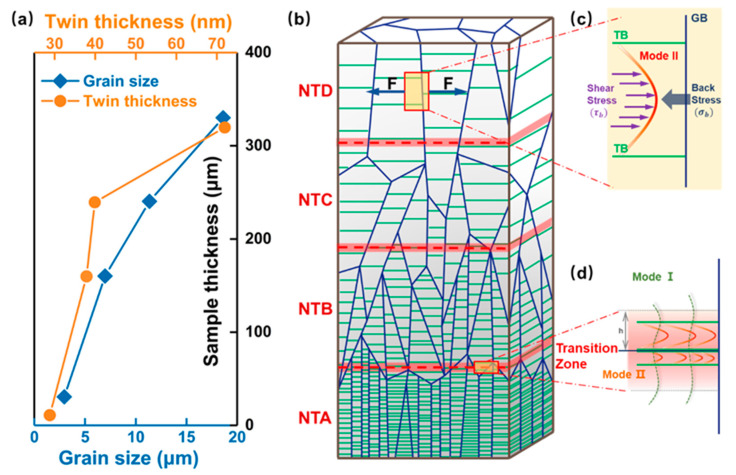
Schematic structure of the gradient nanotwinned (GNT) sample and microscopic deformation mechanisms. (**a**) The practical grain size and twin thickness in the experimental samples. (**b**) A GNT structure composed of four nanotwinned (NT) individual parts each with uniform twin thickness. (**c**) Mode II dislocations accumulate nearby grain boundaries (GBs) under uniaxial tensile. (**d**) Bundles of concentrated dislocations (BCDs) interact in the transition zone. Experimental data come from Ref. [5].

In the present study, we employ the highly oriented coherent twins for the simple but typical deformation prototype. Taking the experimental face-centered cubic pure copper samples as simulated models, the schematic architecture of the sample is shown in Figure 1b. The GNT structure is composed of four NT units which are labeled NTA, NTB, NTC and NTD from bottom to up and each unit contains uniform density of twins with average twin thickness λ. Uniaxial tension is applied parallel to the twin planes to investigate the mechanical response of these samples.

### 2.1. Constitutive Model for Nanotwinned Metals

Before considering the overall multilayer gradient structure metals, we first focus on the homogenous components which are the constituent elements of the GNT metals. When applying a uniaxial loading parallel to the twin planes on the NT metal alone, it comes through elastic section and comes into plastic region with irreversible strain. Each twin lamella is regarded as the perfect elastoplastic material and will yield simultaneously due to the assumed uniform distribution of TBs. Meanwhile, a majority of short dislocation segments with Burgers vectors parallel to the TB will emerge to carry the plastic deformation in the interlayers of twin planes, and we call dislocations with this unique pattern mode II dislocations [37,38]. The justified CLS model is taken to calculate the yield stress, which can be described by [36]:

(1) $$\sigma_{y}\left(\lambda \right)=\sigma_{0}+\beta \frac{\mu b}{\lambda }\ln\left(\frac{\phi \lambda }{b}\right).$$

where $\sigma_{0}$ is the resistance stress associated with lattice friction stress, ϕ and β are material constants associated with dislocation core extension and the Taylor factor, respectively [37], μ is the shear modulus of the material, b is the magnitude of the Burgers vector for the mode II dislocations. It is worth noting that the twin thickness λ of all lamellas is exactly the same in each NT components.

Under the continued loading condition, the large amount of mode II dislocations will move collectively along TBs and ultimately pile up in front of GBs due to the decrease spacing near GBs (see Figure 1c). The corresponding accumulated zone is limited [38] and gives rise to another resistance stress on these model II dislocations which is often called back stress $\sigma_{b}$. The spatial pattern of dislocations is associated with the evolution of the increased plastic stress after yielding. A valid physical expression for such back stress is:

(2) $$\sigma_{b}=M\frac{\mu b}{d_{G}}N$$

where *M* is the Taylor factor, *d_G_* represents the average grain size of samples, *N* is the number of the pile-up dislocations near the GBs and depends on the plastic evolution [38,39,40]. The dependence can be defined by:

(3) $$\frac{dN}{d\varepsilon^{p}}=\frac{\xi }{b}\left(1-\frac{N}{N_{B}}\right)$$

where $\varepsilon^{p}$ is the plastic strain, ξ is the average distance between slip bands and is thought to be proportional with the grain size $\xi =\theta \cdot d_{G}$, $N_{B}$ is the limit for the dislocation sites and represents the finite accumulated zone near the GBs. We find that the accumulated zone will in the saturation of the dislocation storage and therefore the reflected back stress will reach the peak.

Finally, it is necessary to briefly summarize the deformation behaviors for NT metals under uniaxial loading parallel to the twin planes. First of all, elastic deformation is a reversible section. With the increasing loading, all twin lamellas yield at the same time and followed by strengthening contributed by the accumulated dislocations nearby GBs. The true stress in the plastic region can be written as:

(4) $$\sigma =\sigma_{y}\left(\lambda \right)+\sigma_{b}$$

### 2.2. Constitutive Model for Gradient Nanotwinned Metals

As a multilayer gradient structure, a constitutive model is required to elucidate the extra strengthening of GNT metals, rather than simply combining the strength of their constituent elements directly. A physics-based constitutive model for the GNT metals will be formulated that can accurately describe the relationship between the macroscopic gradient structure and the exhibited extra strengthening property. Assisted by the monolithic tension experiment and molecular dynamic simulations [5], the underlying deformation mechanism was achieved. The GNT Cu sample consisted of different components with gradual TB spacings and the loading direction was also oriented in parallel to the twin planes. Although uniform uniaxial tension was acted, the components of the GNT sample with different twin thickness yielded at different stages according to the CLS model, giving rise to unequal plastic strain between neighboring layers. That is the critical difference between NT and GNT metallic materials. Therefore, theoretical analysis conduction needs to be transferred from grain interiors to the extraordinary interfaces among these different components.

We assume a transition zone with the height of h in each NT component where unusual phenomena happen distinguished from that in NT metals. The induced gradient plastic strain from unequal deformation can be defined by [5]:

(5) $$\eta =\frac{\varepsilon_{i+1}^{p}-\varepsilon_{i}^{p}}{h}$$

Here, $\varepsilon_{i+1}^{p}$ and $\varepsilon_{i}^{p}$ are the plastic strain at the edge of the transition zone in the *i*-th gradual interface. Under the circumstances, geometrically necessary dislocations [41,42,43] (GNDs) generate to accommodate the plasticity heterogeneities. The density of GNDs $\rho_{\mathrm{GND}}$ can be given as [5,43]:

(6) $$\rho_{\mathrm{GND}}=\frac{\eta }{b}$$

where *b* is the magnitude of the Burger vector of the GNDs. As demonstrated by Yang et al. [44,45,46,47], pile-up of GNDs lead to the long-range internal stress field in the sample and the stress in the direction perpendicular to the loading direction motivates another dislocation type in the transition zone (see Figure 1d). The emerged dislocations are identified as mode I dislocations with Burgers vectors inclined to the TBs. Model I dislocations are the accommodation for the plastic deformation in highly oriented nanotwins when the loading direction perpendicular to the TBs [48]. Eventually, the interaction between three kinds of dislocations, which are mode I dislocations, mode II dislocations and GNDs, form the bundles of concentrated dislocations (BCDs) and dominates the gradient plasticity in the transition zone. Therefore, that is exactly the strong interaction of BCDs contributing to the extra strengthening in GNT metals.

According to the classical Kocks-Mecking model, the density of the mode I and mode II dislocations, which we denote as $\rho_{I+\mathrm{II}}$, can be calculated through [49]:

(7) $$\frac{\partial \rho_{\text{I}+\text{II}}}{\partial \varepsilon^{P}}=M(\frac{k}{d_{G}}+k_{\text{1}}\sqrt{\rho_{\text{I}+\text{II}}}-k_{\text{2}}\rho_{\text{I}+\text{II}})$$

where *M* is the Taylor factor, k=1/b, $k_{1}=\psi /b$, $k_{2}=k_{20}{\left({\dot{\varepsilon }}^{p}/{\dot{\varepsilon }}_{0}\right)}^{-1/n}$, ψ represents the ratio between the average dislocation distance and the mean free path of gliding dislocations, $k_{20}$ is a material parameter, ${\dot{\varepsilon }}_{0}$ is the reference strain rate, n depends on the temperature, and $\varepsilon^{p}$ is the local plastic strain. According to the Taylor hardening law, the extra strengthening in GNT metals can be quantitatively analyzed through the density of BCDs. The generated extra stress $\sigma_{extra}$ per unit volume in transition zone can be expressed as [50]:

(8) $$\sigma_{extra}=M\alpha \mu b\sqrt{\rho_{I+\mathrm{II}}+\rho_{\mathrm{GND}}}$$

where α is an empirical coefficient. Ultimately, we can obtain the plastic stress of GNT metals through the combination between homogeneous components and the extra elements which depend on their volume fraction.

## 3. Results and Discussion

Based on the proposed theoretical model and experimentally measured parameters [5], we construct a detailed quantitive description of the GNT samples. What we show in Figure 1a is the grain size and twin thickness for the individual NT components. The average twin thickness increases from 29 nm in NTA to 72 nm in NTD and the corresponding average grain size is in the range of 2.5–15.8 μm. To understand the mechanical behaviors thoroughly, we changed the stacking sequence and fabricated the following four spatial distributions while keeping the constant total length: GNT1-“ABCD”, GNT2-“ABCDDCBA”, GNT3-2×“ABCDDCBA”, GNT4-4×“ABCDDCBA”. Except for the structural gradient, the yield stress gradient we calculate from the CLS model also exists in these samples. We define the yield strength gradient, *sg*, as the increment of yield stress per unit length along the gradient direction. All the parameters in the modeling are listed in Table 1.

**Figure 2:**
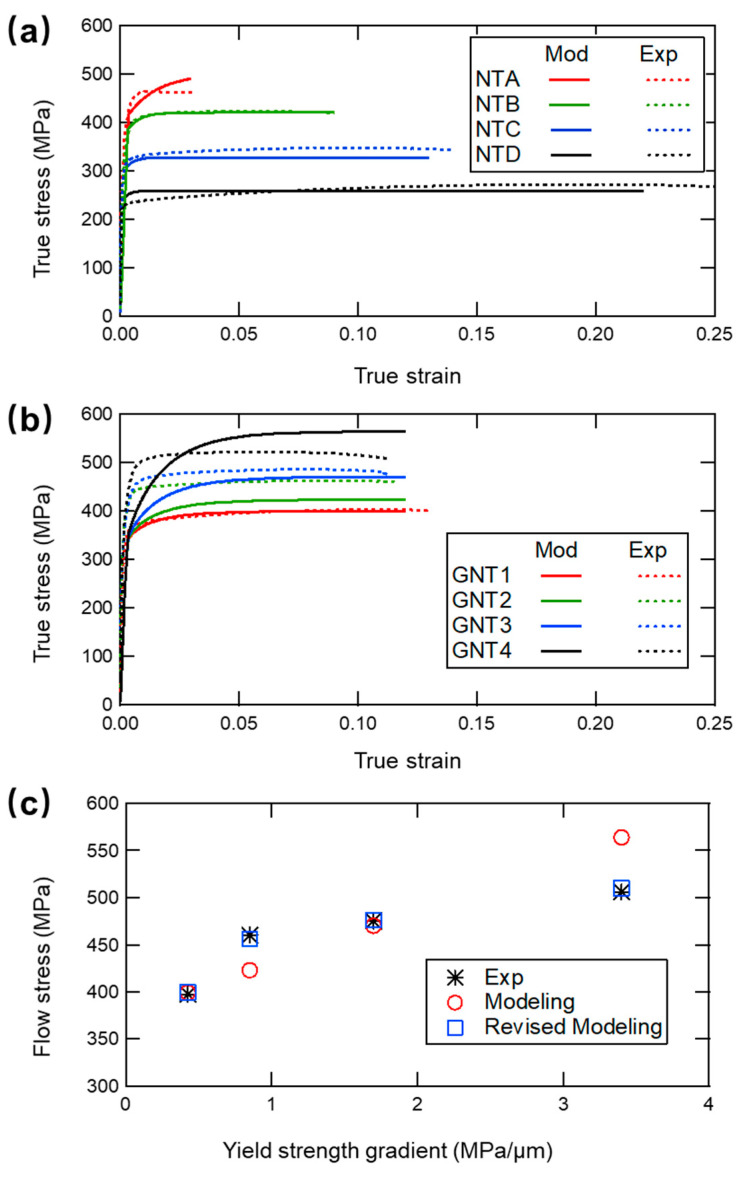
Mechanical properties of samples from modeling results and previous experiment. (**a**) True stress-true strain curves of NT samples. (**b**) True stress-true strain curves of GNT samples. (**c**) Flow stress obtained from experimental, modeling and revised modeling results with various yield strength gradient. Experimental data come from Ref. [5].

We start by comparing the theoretical tensile true stress-true strain relations with those reported in the experiment (see Figure 2a). Nice agreement with experimental results in the plastic flow region can be observed. As characterized by the true stress-true strain curves, we can observe that the plastic stress becomes strain insensitive gradually until ultimate failure happens. The direct cause to the insensitivity is the unchanged back stress superposed on the yield stress when reaching the saturated state in the accumulated zone.

As shown in Figure 2a,b, the extra GNT strengthening is remarkable and even superior to the strongest component in certain GNT structures. In such modeling cases, we assign a constant height, 15 μm, to the transition zone in allsamples. Comparing the mechanical properties in the four GNT samples given in Figure 2c, the flow stress of GNT metals increases gradually with the yield strength gradient. For the perfect elastoplastic GNT samples, the dislocations in the crystal interior become more complex and interact with neighboring dislocations, finally leading to the extra strengthening effect. Compared with the density of mode I and mode II dislocations, the calculated density of the GNDs has a lower magnitude by two orders, meaning that it contributes little to the extra stress according to the Taylor hardening law. However, we cannot neglect the great influences of GNDs on the local stress field among gradient layers, which in turn motivate mode I dislocations in the transition zone.

**Figure 3:**
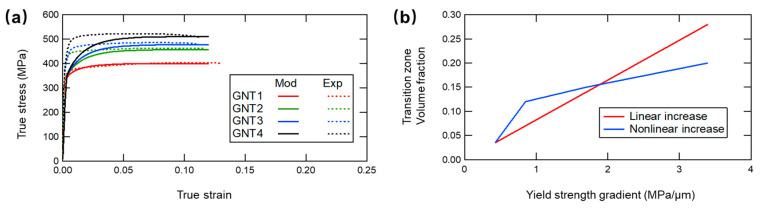
(**a**) the true stress-true strain relation of the GNT samples is calculated according to the ultimate stress in experiment. (**b**) The volume fraction of the transition zone as a function of yield strength gradient with constant height (linear increase) and revised height (non-linear increase). Experimental data come from [5].

In fact, the discrepancy of the ultimate tensile strength except for GNT1 (red curves) in Figure 2b or c is obvious. Characterized by the volume fraction of transition zone in evaluating the extra stress $\sigma_{extra}$ in GNT metals, the constitutive model initially assigns a constant height to all the interaction zone for simplicity. For the sake of grasping the features of the transition zone, we utilize the experimental evolution of extra strengthening and fit with the stress-strain curves to calculate the practical transition zone. We only focus on stability of the flow stress as given in Figure 3a. The total height and the volume fraction of the transition zone are shown in Table 2 and Figure 3b, respectively. These results demonstrate that the linear overlay with the increasing number of the interfaces of these components is not sufficient to predict the mechanical performances correctly. Although the four GNT samples have the same total length, the BCDs near the interfaces between different components are diverse since the unequable length of the individual NT components. The transition zones are distinct and possess non-uniform height among the gradient layers.

Besides, we can observe the descending slope with the gradually increased individual stacking components in Figure 3b (non-linear increase). This may be explained by the finite total length of these samples. Although the spatial distribution of TB layers become more denser, the length of the independent components decreases sharply and it is more likely to generate non-uniform twin thickness in the interfaces for components with sparse TBs in complex GNT structures. Therefore, we need more accurate and reasonable information of the transition zone to seize the actual extra strengthening in the GNT metals, which will be left for our future investigation.

Special attention should be given to the initial transient disagreement between modeling results and experimental measurement (Figure 2b and Figure 3a). The density of the BCDs in transition zone is likely to reach the peak value at the beginning of the plastic deformation due to the unique gradient structure in experiment. The synthetic interfaces between individual components may possess imperfections and the twin thicknesses are non-uniform. These give rise to the rapid increase of the density of BCDs, leading to the more abrupt curves than those of theoretical models. Therefore, a comprehensive model accounting for the evolution of the dislocation density deserves further efforts.

## 4. Conclusions

In conclusion, the present work has developed a physically based theoretical framework to describe the mechanical behaviors of NT and GNT metals under uniaxial tensile. Perfect elastoplastic model and the CLS model are adopted for the homogeneous components in NT metals, and the concept of pile-up mode II dislocations in the finite space near GB, which contributes to the back stress, is adopted in our framework. The model for the extra strengthening effect in GNT metals is based on the microstructural analysis. The gradient deformation among the interfaces gives rise to the formation of GNDs to accommodate the inequal plastic strain among individual components. Mode I dislocations are motivated by the stress field exerted by GNDs, and the strong interaction between mode I and mode II dislocations in the gradient transition zone contributes to the great performance in materials strength, which is the origin of the reported extra strengthening in GNT metals. The constitutive description of the GNT metals is based on the calculated density of the BCDs, which directly reflects the interaction in the transition zone. To sum up, the constitutive model reported in this work can be used to predict the extra strengthening behavior of GNT metals via combining the grain size, twin thickness and the reasonable transition height of the GNT structure quantitatively. It may provide necessary guides for the design and optimization of gradient structural materials in engineering applications.

Our theoretical framework also enlightens several open questions for future studies:

(a) The density of dislocations is a critical basis of the constitutive model for the GNT metals to elucidate the extra strengthening effect. During the early age of the plastic deformation, the density may change suddenly due to the unusual gradient interfaces. The response of the density of the dislocations based on the Kocks-Mecking model may need modification to better accommodate the unusual structure.
(b) The preliminary analysis of the effects about the transition height on the modeling results has been discussed in our present work. The weight of transition height directly influences the calculated stress of the GND metals. Comparing with the non-uniform distribution of the transition zone in the gradient structure, the relation between the height and the gradient interfaces requires further investigations.

## Figures and Tables

**Table 1:** Parameters in modeling.

$\sigma_{0}=65\mathrm{MPa}$	β=0.42	ϕ=0.16	μ=42GPa	b=0.147nm	θ=0.05
M=3	$N_{B}=10$	ψ=0.2	${\dot{\varepsilon }}_{0}=1/s$	n=12.5	α=0.3

**Table 2:** The total height of transition zone in the GNT samples of the original and the revised modeling, respectively.

	GNT1	GNT2	GNT3	GNT4
Initial modeling (μm)	14	28	56	112
Revised modeling (μm)	14	48	60	80

## Data Availability

The data presented in this study are available on reasonable requests from the corresponding author.
